# Factors associated with pre-diabetes in Tehranian men and women: A structural equations modeling

**DOI:** 10.1371/journal.pone.0188898

**Published:** 2017-12-07

**Authors:** Parisa Amiri, Sara Jalali-Farahani, Mehrdad Karimi, Reza Taherian, Sara Kazempour-Ardebili, Firoozeh Hosseini-Esfahani, Parvin Mirmiran, Fereidoun Azizi

**Affiliations:** 1 Research Center for Social Determinants of Health, Research Institute for Endocrine Sciences, Shahid Beheshti University of Medical Sciences, Tehran, Iran; 2 Student Research Committee, Shahid Beheshti University of Medical Sciences, Tehran, Iran; 3 Department of Epidemiology and Biostatistics, School of Public Health, Tehran University of Medical Sciences, Tehran, Iran; 4 Endocrine Research Center, Research Institute for Endocrine Sciences, Shahid Beheshti University of Medical Sciences, Tehran, Iran; 5 Nutrition and Endocrine Research Center, Research Institute for Endocrine Sciences, Shahid Beheshti University of Medical Sciences, Tehran, Iran; International University of Health and Welfare School of Medicine, JAPAN

## Abstract

**Objective:**

To examine associations of sex-specific related factors with pre-diabetes in Tehranian non-diabetic adults.

**Methods:**

This study has been conducted within the framework of the Tehran Lipid and Glucose Study (TLGS) between 2008–2010. A total of 5568 (55.4% female) non-diabetic adults, aged ≥20 years, selected from among participants of the TLGS, were recruited for the study. Data on socio-behavioral factors, family history of diabetes and cardio-metabolic risk factors were included in the hypothesized model to test their direct and indirect associations with pre-diabetes in men and women separately, using structural equation modeling.

**Results:**

Pre-diabetes was diagnosed in 23.6% of participants, with significantly higher prevalence in men compared to women (27.4% and 20.5%, respectively; p<0.001). Body mass index (BMI) and triglycerides (TG) in both sexes and hypertension and high density lipoprotein only in women, were directly associated with pre-diabetes (p<0.05). Poor diet in women was the only behavioral factor directly associated with pre-diabetes (p<0.05). Age in both sexes and education, only in women, were directly associated with pre-diabetes. In both genders, age, marital status, education, employment, poor diet and leisure time physical activity were indirectly associated with pre-diabetes through cardio-metabolic risk factors.

**Conclusions:**

The main modifiable factors directly associated with pre-diabetes were TG in women and BMI in men, which need to be prioritized in health policies for diabetes prevention programs in Tehranian adults. Future research should focus on the gender-specific determinants and underlying mechanisms for TG levels and BMI status among this population.

## Introduction

Similar to other developing countries in the Middle East, Iran is undergoing epidemiological transition and non-communicable diseases, such as diabetes, are the main causes of morbidity and mortality in this country [1]. Between 1980 and 2014, the prevalence of diabetes has more than doubled in the Middle East and Northern Africa (MENA) region, and is predicted to increase substantially in the next 10 years [1,2]. Because diabetes remains a major cause of disability and the fourth-leading cause of fatality in Iran [3,4], it imposes a heavy financial burden on the health-care system [5].

Pre-diabetes is the state in which the blood glucose level of an individual is higher than normal but not high enough to be classified as diabetes [6]. Pre-diabetes imposes a potential burden of mortality on affected individuals [7], which may be related to its association with cardiovascular diseases [8]. Approximately 5–10% of those with pre-diabetes progress to diabetes each year, and with longer observation this progression rate may increase [9]; this is an even more alarming statistic when considering the current high prevalence of pre-diabetes among Iranian adults (18.2%) [10]. Identifying predisposing factors of pre-diabetes in the Iranian population may help delay or prevent diabetes, thereby reducing its mortality and morbidity among this less studied population.

Associations of different behavioral, socio-demographic and cardio-metabolic factors with pre-diabetes have been established in previous studies [11–16]. Physical inactivity and unhealthy diets are the main behavioral factors associated with increased risk of progression to pre-diabetes [11–13]. Among the socio-demographic factors, marital status, higher age and lower education are known predisposing factors of pre-diabetes [11,13–15], while BMI, hypertension, low high density lipoprotein cholesterol (HDL-C) and high levels of triglycerides (TG) are the cardio-metabolic factors shown to have the strongest effects on pre-diabetes [11,13,16].

Since the pre-disposing socio-behavioral and cardio-metabolic factors of pre-diabetes are closely inter-related, considering the mediators of these associations, rather than merely examining their direct associations, could lead to a better understanding of the role of these factors in the development of pre-diabetes. Applying the statistical approach of structural equation modeling (SEM) allows us to conceptualize the structure of predisposing factors of pre-diabetes as a model and simultaneously analyze all relevant regression pathways. Two previous studies have used the SEM method in this field; one looks at type 2 diabetes in a Middle Eastern setting [17] and the other focuses on pre-diabetes in American adults, aged over fifty [18]. Besides, although many gender differences in relation to the predisposing factors of pre-diabetes are reported [19], there is still no study aimed at comparing these factors in men and women separately. Hence, using SEM, this study is the first to investigate sex-specific patterns of the direct and indirect associations of potential socio-behavioral and cardio-metabolic risk factors with pre-diabetes among an adult, Middle-Eastern population.

## Materials and methods

### Subjects and design

The current study was conducted within the framework of the Tehran Lipid and Glucose Study (TLGS), a large scale community based prospective study performed on a representative sample of residents of district-13 of Tehran, the capital of Iran. Details of the rationale and design of the TLGS have been published elsewhere [20,21]. The TLGS has two major components: Phase 1 (1999 to 2001), a cross-sectional prevalence study of non-communicable diseases (NCDs) and their associated risk factors; Phase 2 is an ongoing prospective follow-up study in which NCD risk factors are measured approximately every 3 years. Following baseline data collection, the intervention phase of the study designed to promote healthy lifestyles and prevent NCD risk factors [20].

Data of 5568 (2486 male and 3082 female) non-diabetic participants aged ≥20 years of the TLGS (phase 4, 2008–2010), who had complete data on socio-demographic factors, leisure time physical activity, dietary patterns, family history of diabetes and cardio-metabolic risk factors, were analyzed. Written informed consent was obtained and the study was approved by the ethics committee of the Research Institute for Endocrine Sciences (RIES), Shahid Beheshti University of Medical Sciences.

### Definitions and measures

A trained interviewer collected information on socio-demographic data and family history of diabetes using a validated questionnaire. Information on leisure time physical activity was assessed using the Modifiable Activity Questionnaire (MAQ) [22] and MET-min/day of leisure time activity was calculated. Participants were eventually categorized and then assigned ordinal numbers, corresponding to their levels of activity; those participants who had no leisure time physical activity were categorized in one group and the remaining participants were categorized into four groups, according to quartiles of daily calculated MET for leisure time physical activity. Dietary data was collected using a validated 168-item semi-quantitative food frequency questionnaire (FFQ) [23] by trained dietitians with at least 5 years of experience in food consumption surveys. The participants were asked to report their usual intake (portion size) of each food item during the last year based on a daily, weekly, monthly and yearly basis and these portion sizes were then converted to daily intakes (gram). Data on 23 food groups were used for detecting dietary patterns using the Exploratory Factor Analysis (EFA) method [24]. Anthropometric data including weight and height of participants were measured according to standard protocols and body mass index (BMI) was calculated as weight (kg) divided by the square of height (m2). BMI was categorized into four groups (<18.5, 18.5–24.9, 25–29.9 and ≥30). Blood pressure was measured twice on the right arm after a 15 min rest in a sitting position using a standardized mercury sphygmomanometer (calibrated by the Iranian Institute of Standards and Industrial Researches); mean of the two measurements was considered as the individual’s blood pressure. Hypertension was defined as SBP ≥140 mm Hg or DBP ≥90 mm Hg or taking antihypertensive medications. Serum total cholesterol and triglycerides were measured using enzymatic calorimetric tests with cholesterol esterase and cholesterol oxidase and glycerol phosphate oxidase, respectively. High TG was defined as TG≥ 1.69 mmol/L in both men and women. HDL-C was measured after precipitation of the apolipoprotein B containing lipoproteins with phosphotungistic acid. Low HDL-C was defined as HDL-C< 1.06 mmol/L (men) or HDL-C< 1.29 mmol/L (women). Pre-diabetes was defined as fasting blood glucose ≥5.6 and <7 mmol/L or 2-h post 75 gram glucose load ≥7.8 and <11.1 mmol/L [25]. Other measures including cardio-metabolic risk factors, leisure time physical activity and dietary intake were obtained using the TLGS protocol [20].

### Statistical analysis

**Fig 1:**
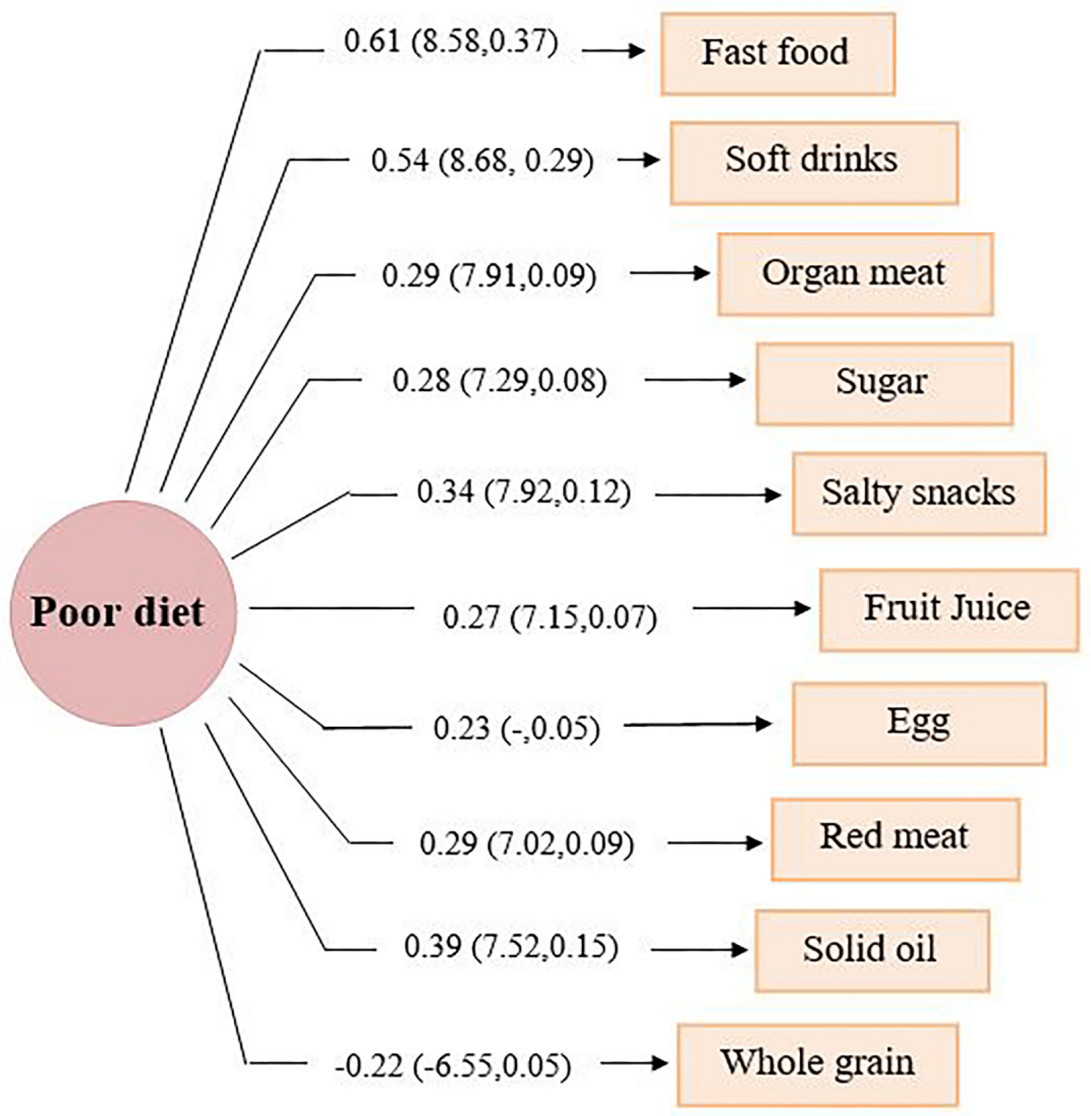
Measurement model of poor dietary pattern: A CFA model based on 50% random sample data (n = 2782). Fit indices of poor diet CFA model: χ^2^ = 34.43, DF = 20, χ^2^/DF = 1.72, RMSEA = 0.016, SRMR = 0.013, GFI = 0.99, CFI = 0.99, IFI = 0.99, NFI = 0.98. The standardized factor loadings (Z-statistics for testing adequacy of explained variance of food groups by poor diet construct, squared multiple correlation of each food group predicted by construct) are reported on pathways.

**Fig 2:**
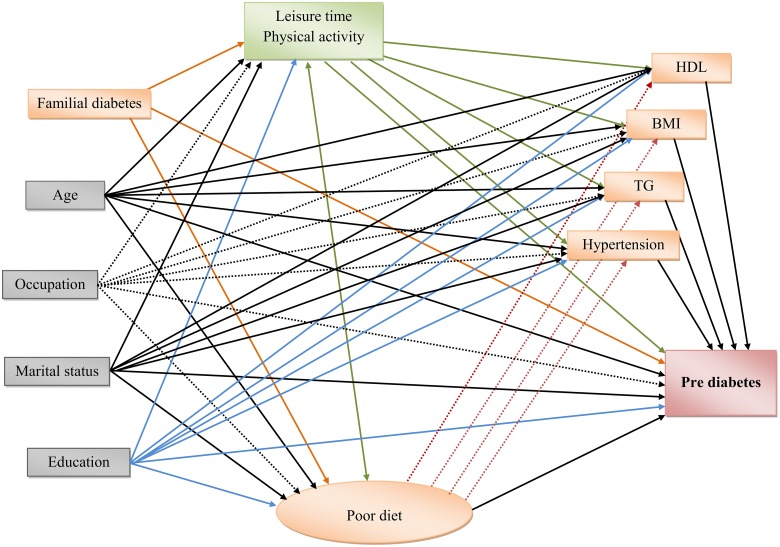
The structural model: Testing the association of socio-behavioral and biochemical factors with pre-diabetes.

Mean±SD for continuous variables and the frequency (%) distribution of categorical data for pre-diabetic and non-pre-diabetic responders have been reported in total and for men and women separately. Independent samples t-test and Chi-Square tests were used to compare continuous and categorical data between those with and without pre-diabetes, respectively. Using Exploratory Factor Analysis (EFA), the latent construct of “poor diet” on 50% randomly selected responders was explored. Principal Component Analysis (PCA) with orthogonal Varimax rotation was conducted to estimate factor loadings which influence responses on observed variables (food groups). Structural Equations Modeling (SEM) was used to test hypothesized patterns of direct and indirect associations among observed and latent variables via the following two-step:

**1**. **The measurement model or CFA**
  In the current study to verify “poor diet” as a latent construct, CFA was used to test the hypothesized associations among a set of food groups and their underlying latent construct(s), which had been already explored by EFA (Fig 1). Food groups with an absolute loading ≥ 0.30 based on EFA remained in the measurement model of poor diet structure.
**2**. **The structural model or SEM**
  In the second stage, the structural modeling approach and path analysis were used to conceptualize inter-correlated variables into a single factor or latent construct (poor diet), and also to explore the direct and indirect associations of behavioral, socio-demographic and cardio-metabolic factors which have been hypothesized to be factors associated with pre-diabetes (Fig 2). Using multiple-group analysis, gender-specific relations between latent and observed variables were also examined. Our focus was on examining the associations of modifiable factors such as physical activity, poor diet, lipids, obesity and high blood pressure on pre-diabetes in each gender separately. The direct associations of non-modifiable confounders including age, sex, level of education, job and marital status are shown in the final model graph; considering the importance of family history of diabetes, this was also examined as a factor of interest. The current SEM models were fitted by the Maximum Likelihood Estimation (MLE) method. All analysis was conducted using IBM SPSS Statistics & Amos version 20. P-values<0.05 were considered statistically significant.

## Results

Mean age of participants was 40.50±13.24 years and pre-diabetes was diagnosed in 23.6% of participants, with a significantly higher prevalence in men compared to women (27.4% and 20.5%, respectively; p<0.001). Table 1 indicates the characteristics of participants according to the presence of pre-diabetes. In both men and women, pre-diabetic individuals had higher mean age, compared to non-pre-diabetic ones. Except for those who had postgraduate degrees, the prevalence of pre-diabetes declined by increasing educational levels. The prevalence of pre-diabetes was significantly higher in married and unemployed participants and in females who had family history of diabetes. In both men and women, higher proportions of pre-diabetic patients did not engage in leisure time physical activity, compared to their non-pre-diabetic counterparts (p<0.01).

**Table 1:** Characteristics of study participants according to the presence of pre-diabetes by genders.

	Men (n = 2486)				P value	Women (n = 3082)				P value
	Normal glucose	95% CI*	Pre-diabetic	95% CI		Normal glucose	95% CI	Pre-diabetic	95% CI	
**Age (years)**	39.17±13.30	-	47.55±13.51	-	<0.001	37.52±11.79	-	47.94±12.96	-	<0.001
**Education**										
Elementary	325(18.0)	16.3–19.8	188(27.7)	24.4–31.1	<0.001	493(20.1)	18.6–21.8	284(44.9)	41.0–48.8	<0.001
Secondary	834(46.2)	43.9–48.5	315(46.3)	42.6–50.1		1177(48.1)	46.1–50.0	254(40.1)	36.4–44.0	
Undergraduate degree	552(30.5)	28.5–32.7	141(20.7)	17.8–23.9		718(29.3)	27.5–31.1	86(13.6)	11.1–16.4	
Postgraduate degree	95(5.3)	4.3–6.4	36(5.3)	3.8–7.2		61(2.5)	1.9–3.2	9(1.4)	0.7–2.6	
**Marital status**										
Single	515(28.5)	26.5–30.6	76(11.2)	9.0–13.7	<0.001	493(20.1)	18.6–21.8	36(5.7)	4.1–7.7	<0.001
Married	1291(71.5)	69.4–73.5	604(88.8)	86.3–91.0		1956(79.9)	78.2–81.4	597(94.3)	92.3–95.9	
**Employment**										
Unemployed	367(20.3)	18.5–22.2	166(24.4)	21.3–27.7	<0.05	1905(77.8)	76.1–79.4	563(88.9)	86.3–91.2	<0.001
Employed	1439(79.7)	77.8–81.5	514(75.6)	72.3–78.7		544(22.2)	20.6–23.9	70(11.1)	8.8–13.7	
**Family history of diabetes**										
No	1662(92.0)	90.7–93.2	614(90.3)	87.9–92.3	0.19	2191(89.5)	88.2–90.6	548(86.6)	83.8–89.1	<0.05
Yes	144(8.0)	6.8–9.3	66(9.7)	7.7–12.1		258(10.5)	9.4–11.8	85(13.4)	10.9–16.2	
**Body Mass Index (Kg/m**^**2**^**)**										
<18.5	29(1.6)	1.1–2.3	1(0.1)	0.0–0.7	<0.001	57(2.3)	1.8–3.0	1(0.2)	0.0–0.7	<0.001
18.5–24.9	654(36.2)	34.0–38.4	137(20.1)	17.3–23.3		859(35.1)	33.2–37.0	89(14.1)	11.5–16.9	
25–29.9	818(45.3)	43.0–47.6	342(50.4)	46.5–54.0		961(39.2)	37.3–41.2	241(38.1)	34.4–41.9	
≥30	305(16.9)	15.2–18.7	200(29.4)	26.1–32.9		572(23.4)	21.7–25.1	302(47.7)	43.8–51.6	
**TG (mmol/L)**										
<1.69	1128(62.5)	60.2–64.7	321(47.2)	43.5–51.0	<0.001	1966(80.3)	78.7–81.8	3331(52.6)	48.7–56.5	<0.001
≥1.69	678(37.5)	35.3–39.8	359(52.8)	49.0–56.5		483(19.7)	18.2–21.3	300(47.4)	43.5–51.3	
**Hypertension**										
No	1277(70.7)	68.6–72.8	396(58.2)	54.5–61.9	<0.001	2061(84.2)	82.7–85.6	403(63.7)	59.9–67.3	<0.001
Yes	529(29.3)	27.2–31.4	284(41.8)	38.1–45.5		388(15.8)	14.4–17.3	230(36.3)	32.7–40.1	
Low HDL** **(mmol/L)**										
No	1079(59.7)	57.5–62.0	346(50.9)	47.1–54.6	<0.001	2129(86.9)	85.6–88.2	460(72.7)	69.1–76.0	<0.001
Yes	727(40.3)	38.0–42.5	334(49.1)	45.4–52.9		320(13.1)	11.8–14.4	173(27.3)	24.0–30.9	
**Leisure Time Physical activity (MET/day)**										
No	779(43.1%)	40.9–45.4	337(49.6%)	45.8–53.3	<0.01	1199(49%)	47.0–50.9	330(52.1%)	48.2–56.0	0.17
Q1	250(13.8%)	12.3–15.5	100(14.7%)	12.2–17.5		308(12.6%)	11.3–13.9	70(11.1%)	8.8–13.7	
Q2	267(14.8%)	13.2–16.5	86(12.6%)	10.3–15.3		334(13.6%)	12.3–15.0	83(13.1%)	10.7–15.9	
Q3	226(12.5%)	11.0–14.1	84(12.4%)	10.0–15.0		341(13.9%)	12.6–15.3	98(15.5%)	12.8–18.5	
Q4	284(15.7%)	14.1–17.5	73(10.7%)	8.6–13.2		267(10.9%)	9.7–12.2	52(8.2%)	6.3–10.5	

*Confidence interval for proportions calculated using Jeffrys method.

**Low HDL-C (female<1.29 and male <1.06)

Poor diet, considered as a latent construct, was extracted using EFA on approximately randomly 50% of the selected participants (n = 2782). Two factors, i.e. 1) healthy dietary patterns and 2) poor dietary patterns explained healthy and unhealthy diets respectively, with total variance being 21.67%, of which 11.58% was explained by the former and the remaining by the latter. The Kaiser-Meyer-Olkin (KMO) index was 0.64. A determinant of 0.021 indicated no co-linearity problem; assumption of sphericity was confirmed using Bartlett's Test (Chi-Square = 10786.51, DF = 253; P<0.001). Varimax rotated factor loadings for healthy and poor diet, extracted using EFA are presented in Table 2. To confirm the measurement model of the poor diet construct, CFA was conducted on the remaining approximately 50% of participants (n = 2786). Fig 1 displays the measurement model for poor diet with observed variables (food groups) which have previously been recognized in EFA. For the measurement model of poor diet, food groups with absolute factor loadings >0.3 were included in the CFA model. Goodness of fit indices reported under Fig 1 indicate acceptable variance explanation and good data fit of the poor diet measurement model.

**Table 2:** Factor loadings for healthy and poor diet extracted using Exploratory Factor Analysis.

	Healthy Diet	Poor diet
Other vegetables	0.76	
Yellow and red vegetables	0.69	
Green vegetables	0.64	
Fruits	0.52	
Refined grains	-0.48	0.22
Rice and Pasta	-0.45	
Liquid oil	0.31	
Low fat	0.29	
Legumes	0.25	
Fish and poultry	0.24	0.22
Fast foods		0.65
Soft drinks	-0.24	0.61
Salty snacks		0.47
Organ meats		0.46
Fruit juice		0.43
Sugar		0.41
Solid oil	-0.23	0.35
Eggs		0.34
Red meat		0.34
Whole grains		-0.32
High fat		0.27
Potatoes		0.20
Tea and Coffee		

**Fig 3:**
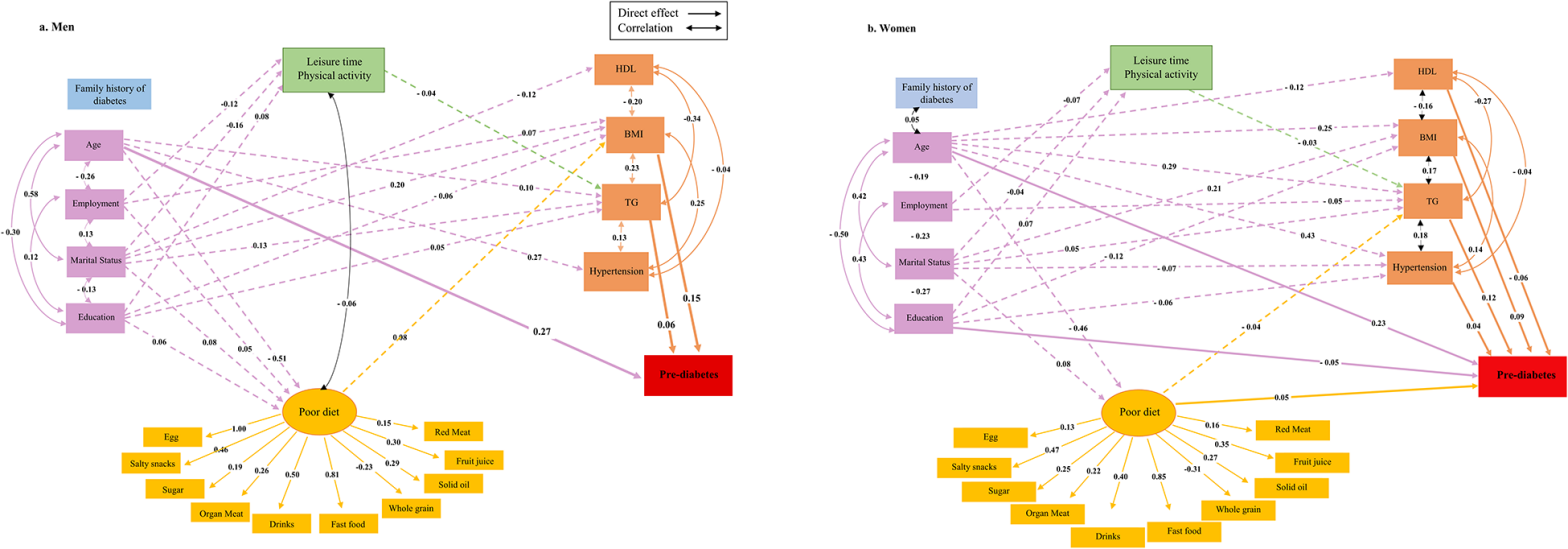
The final structural model after testing the associations of socio-behavioral and biochemical factors with pre-diabetes in (A) Men and (B) Women. Fit indices were acceptable for both SEM models of men (χ2 = 494.87, DF = 114, χ2/DF = 4.34, RMSEA = 0.037, SRMR = 0.029, GFI = 0.98, CFI = 0.94, IFI = 0.94, NFI = 0.92) and women (χ^2^ = 480.41, DF = 114, χ^2^/DF = 4.21, RMSEA = 0.032, SRMR = 0.026, GFI = 0.98, CFI = 0.95, IFI = 0.95, NFI = 0.94).

Hypothesized associations of socio-behavioral and bio-chemical factors with pre-diabetes are depicted in Fig 2. Results of multiple-group analysis show model fit indices for the unconstrained model, in which all parameters differed in men and women, are shown in Table 3. Based on Δχ^2^ = 39.77 which has DF = 14 (P<0.001), the null hypothesis is rejected and results indicate that the unconstrained model fits better than other constrained models which equalize some special parameters in the men and women. Fit indices of SEM models indicate acceptable fit thresholds for both genders and are reported below Fig 3.

**Table 3:** Goodness of fit indices for every pair of nested models and chi-square statistics for comparing the models.

Model	DF	χ^2^	χ^2^ / DF	RMSEA	SRMR	CFI	GFI	NFI	IFI	(Δχ^2^, DF) †
**Unconstrained**	229	981.65	4.20	0.024	0.029	0.94	0.98	0.93	0.94	Assuming to be correct
**Measurement weights**	243	1021.42	4.28	0.024	0.030	0.93	0.98	0.92	0.94	39.77*, DF = 14
**Structural weights**	284	1414.91	4.98	0.027	0.040	0.91	0.97	0.90	0.87	433.26*, DF = 55
**Structural residuals**	299	2331.61	7.80	0.035	0.047	0.85	0.96	0.83	0.78	1349.96*, DF = 70
**Measurement residuals**	346	300.62	8.67	0.075	0.046	0.80	0.93	0.78	0.76	2019.97*, DF = 117

^†^ Chi-square test is used to test the null hypothesis that the more constrained model is correct under the assumption that the less constrained model is correct. The model with all parameters differed in two genders was significantly better than others which had some constraints on the parameters.

* P < 0.001

χ2: Chi-Square value, DF: Degrees of Freedom, RMSEA: Root Mean Square Error of Approximation, SRMR: Standardized Root Mean Square Residual, NFI: Normed Fit Index, CFI: Comparative Fit Index, IFI: Incremental Fit Index, GFI: Goodness of Fit Index.

Table 4 indicates the hypothesized association observed between variables and also the results of the comparison between men and women. Of cardio-metabolic factors studied, pre-diabetes was found to be directly associated with both BMI and TG in both genders (p<0.01) and with hypertension and HDL, only in women (p<0.05). Differences in Z-statistics of the associations between BMI and TG and pre-diabetes were statistically significant among men and women (p<0.05). Poor diet in women was the only behavioral factor which was directly associated with pre-diabetes (β = 0.051, p<0.05). In men, pre-diabetes had indirect associations with both poor diet and physical activity via BMI and TG respectively; in women, these indirect associations between behavioral factors and pre-diabetes were via TG. Leisure time physical activity and poor diet were significantly correlated, only in men (r = -0.056, p<0.01). Among socio-demographic factors, age in both genders (p<0.01) and education only in women (p<0.05) showed direct associations with pre-diabetes. All other socio-demographic factors were found to be indirectly associated with pre-diabetes in both genders (Fig 3).

**Table 4:** Sex-specific associations between socio-behavioral and biochemical factors and pre-diabetes.

Predictor	Response	Men		Women		Difference
		Coefficient	C.R	Coefficient	C.R	C.R^#^
Age (years)	**Physical Activity**	-0.039	-1.44	-0.007	-0.31	0.89
Employment		-0.123	-5.79**	-0.068	-3.42**	1.93
Marital Status		-0.163	-6.47**	-0.043	-2.13*	3.51**
Education		0.083	4.10**	0.071	3.21**	-0.35
Family history of diabetes		0.028	1.46	0.000	0.40	-1.43
Age (years)	**Poor diet**	-0.506	-7.17**	-0.457	-5.80**	1.00
Employment		0.052	2.28*	0.028	1.42	-1.02
Marital Status		0.077	2.80**	0.076	3.33**	-0.21
Education		0.056	2.58**	0.009	0.40	-1.70
Family history of diabetes		-0.012	-0.58	0.022	1.24	1.15
Physical Activity		-0.056^¥^	-2.34**	0.010^¥^	0.53	2.24*
Age (years)	**HDL**	-0.020	-0.65	-0.118	-4.98**	-2.11*
Employment		0.010	0.45	0.025	1.25	0.33
Marital Status		-0.117	-4.46**	-0.029	-1.47	2.97**
Education		-0.026	-1.23	0.039	1.75	2.06*
Physical Activity		0.018	0.85	0.016	0.88	-0.22
Poor diet		-0.022	-0.85	-0.008	-0.38	0.46
Age (years)	**BMI**	-0.007	-0.24	0.249	11.78**	7.82**
Employment		0.072	3.32**	-0.009	-0.49	-2.76**
Marital Status		0.203	7.89**	0.207	11.59**	1.60
Education		-0.060	-2.91**	-0.123	-6.21**	-2.69**
Physical Activity		0.022	1.10	0.025	1.57	0.24
Poor diet		0.081	2.84**	-0.025	-1.30	-2.96**
Age (years)	**TG**	0.100	3.33**	0.292	13.08**	4.96**
Employment		0.042	1.91	-0.047	-2.49*	-3.04**
Marital Status		0.132	5.11**	0.045	2.36*	-2.74**
Education		0.048	2.31*	-0.019	-0.91	-2.30*
Physical Activity		-0.039	-1.96*	-0.033	-1.97*	0.35
Poor diet		0.021	0.82	-0.044	-2.06*	-1.99*
Age (years)	**Hypertension**	0.274	9.26**	0.425	19.79**	3.49**
Employment		0.014	0.67	-0.012	-0.66	-0.93
Marital Status		0.022	0.86	-0.067	-3.66**	-2.78**
Education		-0.004	-0.19	-0.061	-3.03**	-1.88
Physical Activity		0.034	1.71	-0.003	-0.19	-1.49
Poor diet		0.022	0.88	-0.011	-0.57	-1.04
Age (years)	**Pre Diabetes**	0.268	9.03**	0.233	9.70**	-0.95
Employment		-0.002	-0.10	-0.022	-1.17	-0.66
Marital Status		-0.016	-0.63	-0.023	-1.19	-0.23
Education		-0.029	-1.43	-0.049	-2.37*	-0.66
Family history of diabetes		0.036	1.91	0.030	1.78	-0.55
HDL (mg/dl)		-0.014	-0.67	-0.056	-3.15*	-1.85
BMI (kg/m^2^)		0.151	7.31**	0.093	4.86**	-2.85**
TG(mg/dl)		0.055	2.58**	0.115	5.97**	2.19*
Hypertension		0.001	0.07	0.042	2.22*	1.5
Physical Activity		-0.029	-1.48	-0.006	-0.37	0.93
Poor diet		0.037	1.44	0.051	2.33*	0.57

* P < 0.05,

** P < 0.01,

^#^ Critical Ratio for Difference between Men and Women,

^¥^ Correlation Coefficient.

## Discussion

The current study examined a conceptual model of direct and indirect associations of socio-behavioral and cardio-metabolic factors with pre-diabetes among TLGS men and women. Our results show that age is the only common factor with a marked significant direct relationship with pre-diabetes in both genders, while BMI status and the level of TG are the next strongest factors directly associated with pre-diabetes in men and women, respectively. Other factors directly associated with pre-diabetes include BMI, level of HDL, hypertension, educational level and diet quality in women and TG in men, which all have significant, but weak, associations.

Our results show BMI in men and TG in women to be the most effective modifiable factors that are directly associated with pre-diabetes. The stronger association of BMI with pre-diabetes in men may be related to differences in fat distribution patterns in both genders. Men are more susceptible to central obesity, which has a known detrimental effect on metabolism and is a stronger predictor of pre-diabetes compared to BMI [25,26]. The only other SEM modeling study on pre-diabetes determinants, conducted by Bardenheier et al considered the more accurate determinant of WC instead of BMI as a predisposing factor in their model [18]. However, there are studies that show a similar sex-specific predicting value for BMI in type 2 diabetes, such as the Roche et al study, which reported that being overweight is positively associated with diabetes only in men [27] and Njolstad et al who showed BMI to be the dominant risk factor for developing diabetes in men [28].

In the current study, although elevated TG levels were more prevalent in men, it was a stronger direct predictor of pre-diabetes in women, a finding that contrasts with previous reports which show that in the pre-diabetic state, high TG and low HDL-C are more prevalent among women compared to men [29,30]; suggesting the presence of a sex-specific underlying mechanism for increasing both TG levels and its different detrimental effects on the progression of pre-diabetes among men and women; these mechanisms may include the effect of female hormones, especially estrogen, on insulin resistance pathways [31].

Regarding the behavioral factors of diet and physical activity, we only observed a direct, significant but weak association between poor diet and pre-diabetes in women. Even the significant indirect behavioral associations via TG in women and BMI and TG in men were not considerable. Although these findings show a sex-specific pattern for the association between behavioral factors and pre-diabetes, because of the overall weak association, it undermines the previously hypothesized pivotal role of these factors in the current study. The sex-specific pattern found in our study is in agreement with the results of McNaughton et al, which show that diet quality is associated with the risk of pre-diabetes in women, but not in men [32]. A similar indirect association between diet quality and pre-diabetes was revealed in the Bardenheier et al study, through WC and TG [18]; however, the fact that their study was not sex-stratified restricts its comparison with our results. There is also evidence showing a weak indirect effect of diet on type 2 diabetes via TG among Qatari adults [17]. Based on the current results, physical activity weakly affects pre-diabetes via TG in both genders, consistent with a previous report from an Iranian population in which physical activity was not associated with the risk of pre-diabetes and diabetes [11]. However, the indirect effects of physical activity on pre-diabetes, reported by Bardenheier et al, were stronger and it was via all included cardiovascular risk factors viz. WC, HDL-C, TG and blood pressure [18].

Except for age, the current study demonstrates indirect associations between socio-demographic factors and pre-diabetes in men, which are mediated through TG for age, BMI for employment and both TG and BMI for marital status and education. On the other hand, in women, in addition to age, education is also a direct but weak determinant of progression to pre-diabetes. In addition, the structural model for women shows a more complex indirect associations among all behavioral and cardio-metabolic factors. Similarly, a recent study conducted on Malaysian adults showed age to be the only significant determinant of cardiovascular risk factors in men, whereas age, employment status and education level were all predisposing factors in women [33]. Another study showed that Korean women with low levels of education are at a higher risk for developing diabetes, a finding not observed in men [34]. Women with low education levels tend to have higher levels of psychosocial stress, compared to men in the same category [35], which in turn increases the risk of abnormal glucose metabolism [36].

Despite the fact that a family history of diabetes has repeatedly been identified as a risk factor for pre-diabetes in various studies [18,37] our study did not show any direct or indirect association for family history with the risk of pre-diabetes. Another study from Iran revealed that although a positive family history of diabetes is related to the progression of pre-diabetes to diabetes, it is not a significant risk for the development of pre-diabetes [11].

As for strengths, to the best of our knowledge this is the first study investigating the sex-specific pathways of interrelated factors leading to pre-diabetes among a large Middle-Eastern population. Applying SEM analysis allowed us to assess the direct and indirect associations of socio-behavioral and cardio-metabolic factors on pre-diabetes in a multifactorial model. The results of the present study should however be interpreted in the light of certain limitations. First, the urban population of the current study does not necessarily reflect similar results in rural populations. Second, there are other predisposing factors that could affect pre-diabetes, including income levels and psychological factors such as depression and anxiety, which have not been considered in the current study. Another limitation of our study is the relatively low variance for the two dietary patterns defined, which also has been a common issue in similar studies and is due to the wide variation of food groups [38,39].

In conclusion, our results showed a sex-specific network of associations among potential predisposing factors of pre-diabetes in Tehranian adults. The most significant modifiable factors identified were TG in women and BMI in men, which need to be targeted in health policy strategies for diabetes prevention. Future research should focus on the gender-specific determinants and underlying mechanisms for TG level and BMI status among this population.

## Supporting information

S1 Minimal dataset (SAV)

S1 SEM (AMW)
